# Hypertonic Saline Reduces Vascular Leakage in a Mouse Model of Severe Dengue

**DOI:** 10.1371/journal.pone.0061621

**Published:** 2013-04-18

**Authors:** Grace Kai Xin Tan, Jowin Kai Wei Ng, Kar Wai Tan, Veronique Angeli, Shabbir Moochhala, Eng Eong Ooi, Sylvie Alonso

**Affiliations:** 1 Department of Microbiology, Immunology Programme, Yong Loo Lin School of Medicine, Life Sciences Institute, National University of Singapore, Singapore, Singapore; 2 DSO National Laboratories, Singapore, Singapore; 3 Progamme in Emerging Infectious Diseases, Duke-NUS Graduate Medical School, Singapore, Singapore; Commissariat a l’Energie Atomique(cea), France

## Abstract

Dengue (DEN) is a mosquito-borne viral disease and represents a serious public health threat and an economical burden throughout the tropics. Dengue clinical manifestations range from mild acute febrile illness to severe DEN hemorrhagic fever/DEN shock syndrome (DHF/DSS). Currently, resuscitation with large volumes of isotonic fluid remains the gold standard of care for DEN patients who develop vascular leakage and shock. Here, we investigated the ability of small volume of hypertonic saline (HTS) suspensions to control vascular permeability in a mouse model of severe DEN associated with vascular leakage. Several HTS treatment regimens were considered and our results indicated that a single bolus of 7.5% NaCl at 4 mL per kg of body weight administered at the onset of detectable vascular leakage rapidly and significantly reduced vascular leak for several days after injection. This transient reduction of vascular leakage correlated with reduced intestine and liver damage with restoration of the hepatic functions, and resulted in delayed death of the infected animals. Mechanistically, we showed that HTS did not directly impact on the viral titers but resulted in lower immune cells counts and decreased systemic levels of soluble mediators involved in vascular permeability. In addition, we demonstrated that neutrophils do not play a critical role in DEN-associated vascular leakage and that the therapeutic effect of HTS is not mediated by its impact on the neutrophil counts. Together our data indicate that HTS treatment can transiently but rapidly reduce dengue-associated vascular leakage, and support the findings of a recent clinical trial which evaluated the efficacy of a hypertonic suspension to impact on vascular permeability in DSS children.

## Introduction

Dengue (DEN) is an arthropod-borne viral disease that is endemic in subtropical and tropical countries. With approximately half the world’s population residing in DEN endemic regions and more than 50 million new infections projected to occur annually, DEN certainly poses as a global health threat and economical burden [1]. There is currently no licensed DEN antiviral or vaccine available.

DENV infection in human can be asymptomatic or ranges from mild acute febrile illness associated with the classical DEN fever (DF) to severe DEN hemorrhagic fever/DEN shock syndrome (DHF/DSS). In DHF/DSS patients, capillary leakage develops rapidly over a period of hours at the time of defervescence [2]. Plasma leakage resulting from increased systemic vascular permeability is the most severe and life threatening complication of DENV infection, and can quickly progress to shock if volume loss is not remedied with proper fluid therapy [3].

Currently, resuscitation with isotonic fluid such as Ringer’s lactate (RL) and normal saline (0.9% NaCl) remains the gold standard of care for DEN shock and involves the administration of large volumes of fluid at various rate and over several hours period to restore microvascular perfusion [3], [4]. This approach requires close and frequent monitoring of the patient’s condition to prevent excessive fluid accumulation in the tissues which constitutes one of the major complications of DSS that contributes to mortality [5].

As an alternative strategy to the current fluid resuscitation approach, we investigated whether treatment with a small volume of hypertonic saline (HTS) solution may be beneficial to dengue patients that experience shock. The clinical application of HTS has been described for severe hypovolemia and shock almost two decades ago [6] and, compared to administration of isotonic fluids presents the advantage to involve a much smaller resuscitation volume (3–6 mL/kg), which leads to rapid correction of cardiovascular function and hemodynamics without excessive fluid accumulation [7]. Restoration of normal intravascular physiology by HTS is achieved through a potent transcapillary osmotic gradient that results in the rapid mobilization of fluid from the intracellular compartment to the intravascular space [8]. In addition to serve as a plasma volume expander, HTS has also been reported to affect the activation status and thus function of a number of immune cells including monocytes, polymorphonuclear neutrophils (PMNs) and T-lymphocytes [9]–[15].

In this work, we explored the ability of HTS to reduce vascular permeability in a mouse model of severe DEN associated with vascular leakage. Our study demonstrates that a single bolus of HTS rapidly and significantly reduces vascular leakage for several days, and supports the conclusions of a recent clinical trial which evaluated the efficacy of a hypertonic suspension to impact on vascular permeability in DSS children [16].

## Materials and Methods

### Ethics Statement

All the animal experiments were carried out under the guidelines of the National Advisory Committee for Laboratory Animal Research (NACLAR) in the AAALAC-accredited NUS animal facilities (http://nus.edu.sg/iacuc/). NUS has obtained a license (#VR008) from the governing body Agri-Food & Veterinary Authority of Singapore (AVA) to operate an Animal Research Facility. The animal experiments described in this work were approved by the IACUC from National University of Singapore under protocol number 009/09. Non-terminal procedures were performed under anesthesia, and all efforts were made to minimize suffering.

### Virus Strain and Culture Conditions

The D2Y98P-PP1 virus is a double plaque-purified DENV-2 strain [17], and has been propagated in C6/36 cells (ATCC# CRL-1660) in Leibovitz’s L-15 medium (GIBCO) supplemented with 5% fetal calf serum (FCS) as described previously [18].

### Plaque Assay

Plaque assay was carried out in BHK-21 cells as described previously [19]. Briefly, 2×10^5^ BHK-21 cells were seeded in 24-well plates (NUNC, NY, USA). BHK-21 monolayers were infected with 100 µl of ten-fold serial dilutions of virus suspension [10^−1^ to 10^−8^ in RPMI 1640 (GIBCO)]. After 1 hr incubation at 37°C and 5% CO_2_, 1 ml of 1% (w/v) carboxymethyl cellulose in RPMI supplemented with 2% FCS was added. After 4 days incubation, the cells were fixed with 4% paraformaldehyde and stained with 1% crystal violet dissolved in 37% formaldehyde. After thorough rinsing with water, the plates were dried and the plaques were scored visually and expressed as the number of plaque forming units (pfu). Triplicates were run for each dilution sample. The limit of detection was set at 10 pfu per ml or per g of tissue.

### Mice Infection with DENV

AG129 breeders [129/Sv mice deficient in both alpha/beta (IFN-α/β) and gamma (IFN-γ) interferon receptors] were obtained from B&K Universal (UK). They were housed under specific pathogen-free conditions in individual ventilated cages. Eight to nine week-old mice were administered with 10^4^ pfu of D2Y98P-PP1 via the subcutaneous (sc) route (0.1 ml in sterile PBS) as described previously [20]. The clinical symptoms were scored as follows: 0 - at healthy state, 1 - signs of ruffled fur, 2 - hunched back, 3–severe diarrhea, 4 - moribund stage, 5 - severe weight loss. The infected animals were monitored daily (clinical score between 0–2) and every 12 hours (clinical score from 3 onwards). Survival rate was derived from the number of mice that were euthanized at moribund stage as evidenced by severe diarrhea, lethargy, and sharp body weight loss as described previously [19], [20].

### HTS Treatments

Mice were intravenously administered at a dose of 4 ml/kg of body weight with either one or three consecutive doses of sterile 5% or 7.5% NaCl suspensions at the indicated time points post-infection.

### Viral Titers in Blood and Organs

The levels of infectious virus in the plasma and tissues from infected mice were assessed as described previously [19]. Briefly, the animals were euthanized and perfused systemically with sterile PBS. Whole tissues and organs were harvested from individual mice and homogenized. Ten-fold serial dilutions of each homogenate (from neat to 1: 10^4^) were assayed by plaque assay as described above. Triplicates were run for each dilution sample. Data are expressed as log_10_ [mean ± SD] in pfu per gram of wet tissue with a limit of sensitivity set at 1.0 log_10_ pfu/g of tissue. Five mice per time point per group were assessed. Results are representative of at least two independent experiments.

### Vascular Leakage

Vascular leakage was determined using the Evans Blue extravasation assay as described previously [19]. Briefly, Evans blue dye (0.5% w/v in PBS) (Sigma Aldrich) was injected intravenously to the anesthetized mice and after 2 hrs, the animals were euthanized and extensively perfused with sterile PBS. The tissues were then harvested and weighed prior to dye extraction with N,N-dimethylformamide (Sigma), and absorbance reading at 620 nm. Data are expressed as fold increase in OD_620nm_ per g of tissue wet weight compared to the uninfected control.

### Histology

Mice were euthanized, and tissues were harvested and immediately fixed in 10% formalin in PBS. Fixed tissues were paraffin embedded, sectioned and stained with Hematoxylin and Eosin (H&E).

### Hematology

Mouse blood samples were collected in K2EDTA and serum tubes (Biomed Diagnostics). Whole blood was immediately analysed for cell counts using automated hematology ***analyzer*** Cell Dyn –3700 ***(***Abbott). Serum alanine (ALT) and aspartate (AST) aminotransferases, albumin total protein, sodium, chloride and creatinine levels were quantified using chemistry analyzer COBAS C111 (ROCHE).

### Detection of Soluble Mediators

Serum levels of various cytokines and other soluble factors were measured using individual detection kits (R&D), according to the manufacturer’s instructions.

### Flow Cytometry

Mouse blood was subjected to red blood cells lysis and resuspended in MACS buffer for antibodies staining. Anti-Ly6G (BD Biosciences) antibody was used to identify the neutrophil population. Neutrophil activation was assessed using rat anti-mouse CD18 detected with anti-rat-APC and PerCPCy5.5-conjugated anti-CD62L (BD Biosciences). For detection of VEGF-A and TNF-α production, surface antigen staining was performed prior to intracellular staining for VEGF-A or TNF-α using the BD Fixed/Permeabilization Kit®, and rabbit purified anti-VEGF-A (Santa Cruz) antibody revealed with APC-Cy7-conjugated anti-rabbit IgG (Santa Cruz) or FITC-conjugated TNF-α antibody (Biolegend). FACS analysis was performed using a CyAn ADP Analyzer (Beckman Coulter) and data were analyzed with Flowjo software (Treestar).

### Neutrophil Depletion

Groups (n = 5) of DENV-infected AG129 mice were injected twice intraperitoneally at day 5 and 6 p.i., with anti-neutrophil antibody NIMP-R14 at 250 µg per mouse [21]. Uninfected mice also received twice NIMP-R14. A control group of 5 infected mice were iv. injected with a bolus of 7.5% NaCl (4 ml/kg) at day 6 p.i. Mice were either monitored daily for their survival and clinical symptoms or sacrificed at day 7 p.i. to assess the activation status of neutrophils, cell numbers, cytokine levels and vascular leakage.

### Statistics

All statistical analysis was done with GraphPad Prism Version 5.0 (GraphPad Software, San Diego, CA, USA). Survival rates were plotted and analyzed according to the Kaplan Meier’s method. Other data were analyzed using two-tailed Student’s *t* test and *p* values <0.05 were considered significant.

## Results

### A Single Bolus of 7.5% NaCl Reduces Vascular Leakage

**Figure 1:**
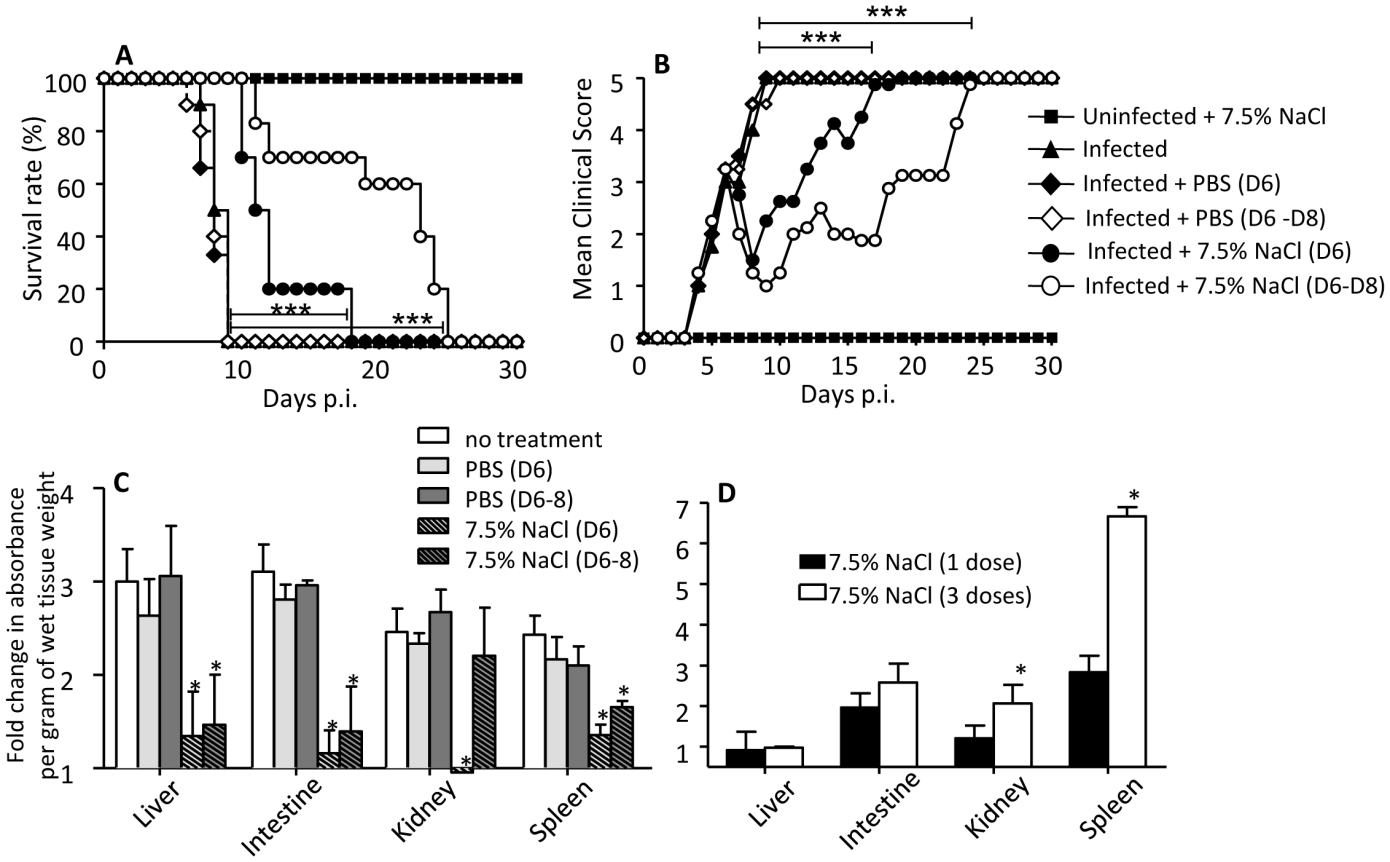
Survival rate, clinical score and vascular leakage in DENV-infected mice treated once or three times with 7.5% NaCl. Groups of 8–9 week-old AG129 mice were sc. infected with D2Y98P-PP1 strain. At day 6 p.i., they were iv. administered with a volume (4 ml/kg body weight) of sterile 7.5% NaCl (HTS) or 0.9% NaCl (PBS) once (D6), or once for three consecutive days (D6–D8) as indicated. (A) Survival rate (n = 10). (B) Clinical scores (n = 10): 0: Healthy; 1: Ruffled fur; 2: Hunched back; 3: Severe Diarrhea; 4: Moribund stage; 5: Severe weight loss. (C) Systemic vascular leakage in infected mice. Three hours post-HTS or PBS treatment, DENV-infected mice (n = 5) were assessed for vascular leakage and the quantity of Evan’s blue dye was determined in various organs from the individual mice as indicated. Results are expressed as fold change compared to uninfected untreated control mice. (D) Systemic vascular leakage in uninfected mice. Uninfected mice (n = 5) were subjected to HTS treatment (once or once daily for three days) followed by Evan’s blue assay 3 hrs after the last administration.

Adult AG129 mice were sc. infected with the D2Y98P-PP1 strain as described previously [20]. In this mouse model, vascular leakage is detectable as early as day 6 post-infection (p.i.) and is much pronounced at moribund stage (day 9–10 p.i.) [20]. To test the effect of HTS treatment on vascular leakage, mice were intravenously (iv.) administered at day 6 p.i. once or once daily for 3 consecutive days (day 6–8 p.i.) with a suspension of 7.5% NaCl at 4 mL/kg body weight or PBS. The survival rates indicated that whereas untreated or PBS-treated infected mice died uniformly by day 10 p.i., HTS treatment significantly delayed the death of the infected animals with the regimen of a daily administration for 3 consecutive days more effective than a single bolus given at day 6 p.i. (Fig. 1A). Concurrently, HTS treatment significantly but transiently improved the clinical scores whereby the animals looked healthy with alleviation of the diarrhea and hunched back symptoms (Fig. 1B). However, 3–4 days post-HTS treatment, the mice displayed body weight loss and signs of lethargy that further progressed into moribund state (Fig. 1B). Consistent with the survival rates, one administration for 3 consecutive days was more potent than a single bolus at delaying occurrence of moribund signs. None of the uninfected control mice died or displayed any clinical manifestations upon HTS treatment.

Systemic vascular leakage was assessed 3 hours post-HTS treatment by Evan’s blue extravasation as described previously [19], [20]. Expectedly, the untreated or PBS-treated infected animals displayed significant increase in systemic vascular permeability which resulted in increased amounts of Evan’s blue dye in the organs tested (Fig. 1C). In contrast, the amount of dye measured in the HTS-treated animals was significantly reduced (Fig. 1C). However, high amount of dye was measured in the kidneys from the animals treated with a daily dose for 3 consecutive days (Fig. 1C). This latter observation suggested that such HTS treatment regimen may cause kidney damage and/or dysfunction. Consistently, uninfected mice that received HTS once daily for 3 consecutive days displayed significant increase in vascular permeability in the kidneys and spleen compared to a single bolus (Fig. 1D), thus suggesting that repeated treatment may not be safe and appropriate for human use.

Altogether, the data indicated that a single-dose iv. administration of 7.5% NaCl at day 6 p.i. is able to reduce DEN-associated vascular leakage without organ (kidney and spleen in particular) damage or dysfunction.

### A Single Bolus of 5% NaCl is Less Effective than 7.5% NaCl at Controlling Vascular Leakage

**Figure 2:**
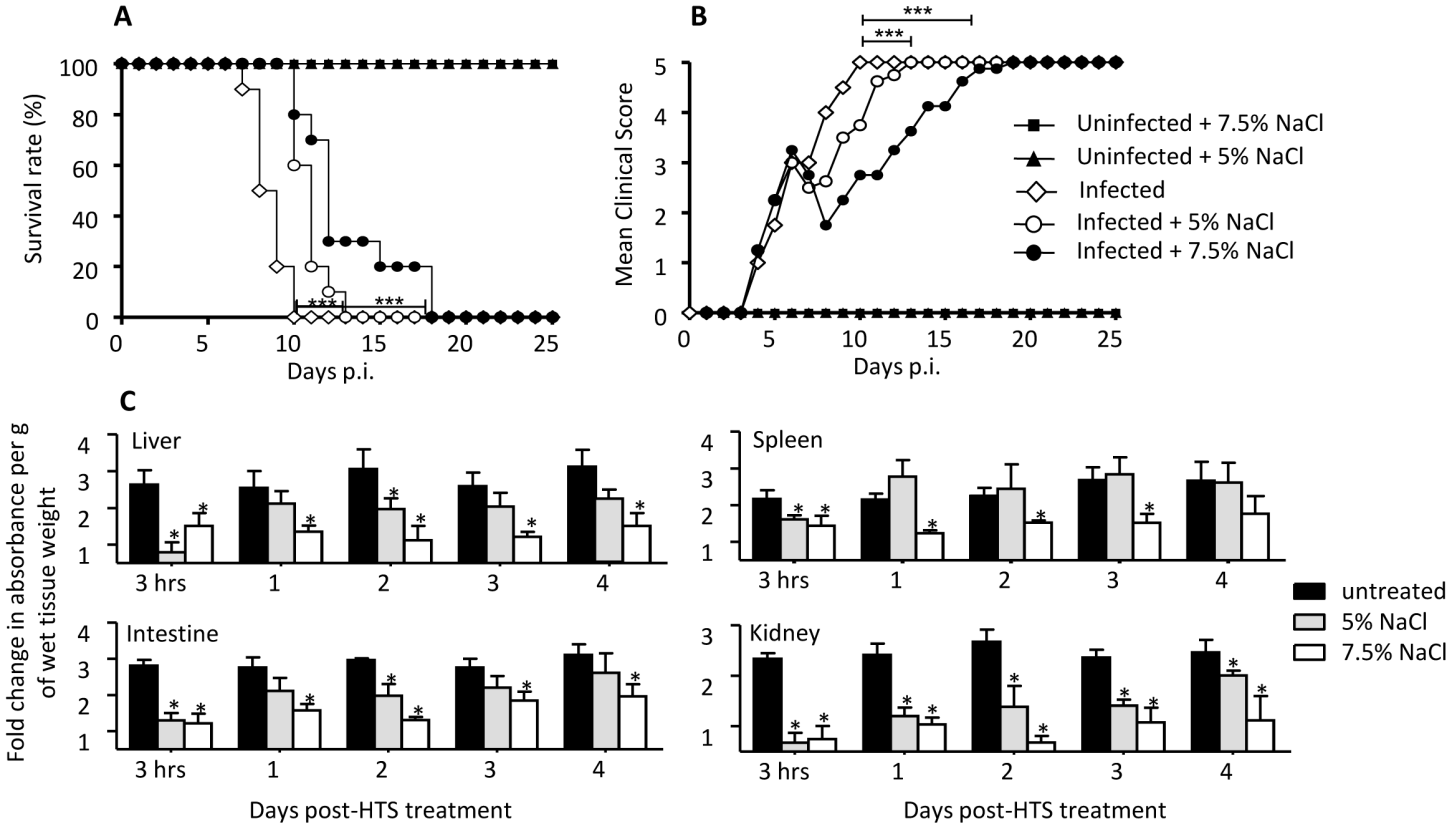
Survival rate, clinical score and vascular leakage in DENV-infected mice treated once with 7.5% or 5% NaCl at day 6 post-infection. Groups of 8–9 week-old AG129 mice were sc. infected with D2Y98P-PP1 strain or left uninfected as indicated. At day 6 p.i., they were iv. administered with a single bolus (4 ml/kg body weight) of sterile 5% or 7.5% NaCl. (A) Survival rate (n = 10). (B) Clinical scores (n = 10) see legend in Fig. 1. (C) Vascular leakage. At the indicated time points post-treatment, the animals were assessed for vascular leakage and the level of Evan’s blue extravasation was quantified in various organs (as indicated) from 5 individual mice per time point. Results are expressed as fold change compared to uninfected untreated control mice. *p<0.05.

To investigate whether a lower salt concentration would result in similar effects, DENV-infected mice were administered iv. with a single bolus of 5% NaCl at day 6 p.i. and the survival and clinical score were determined and compared to those observed with DENV-infected mice treated with 7.5% NaCl. The results indicated that 5% NaCl treatment significantly delayed the death of the animals but not as effectively as 7.5% NaCl treatment (Fig. 2A). Consistently, alleviation of the clinical manifestations in the 5% NaCl-treated animals was not as pronounced and sustained as that observed with the 7.5% NaCl-treated animals (Fig. 2B). Vascular leakage was assessed in various organs at 3 hrs post-HTS treatment, and daily for 4 days. The results indicated that 5% NaCl treatment significantly reduced vascular leakage in the organs assessed but not as effectively as 7.5% NaCl treatment and in a more transient manner, whereby reduction in vascular leakage could be observed only at 3 hrs post-treatment in the spleen, liver and intestine from the 5% NaCl treated-mice whereas reduced leakage was still observed at 3 or 4 days post-treatment in the 7.5% NaCl treated-animals (Fig. 2C). Thus these experiments demonstrated that a single bolus of 7.5% NaCl given at day 6 p.i. is more effective than 5% NaCl to reduce vascular leakage over a longer period of time. Thus the subsequent experiments have been performed using a suspension of 7.5% NaCl (HTS).

### HTS Treatment at a Later Stage of the Disease is Less Effective at Controlling Vascular Leakage

**Figure 3:**
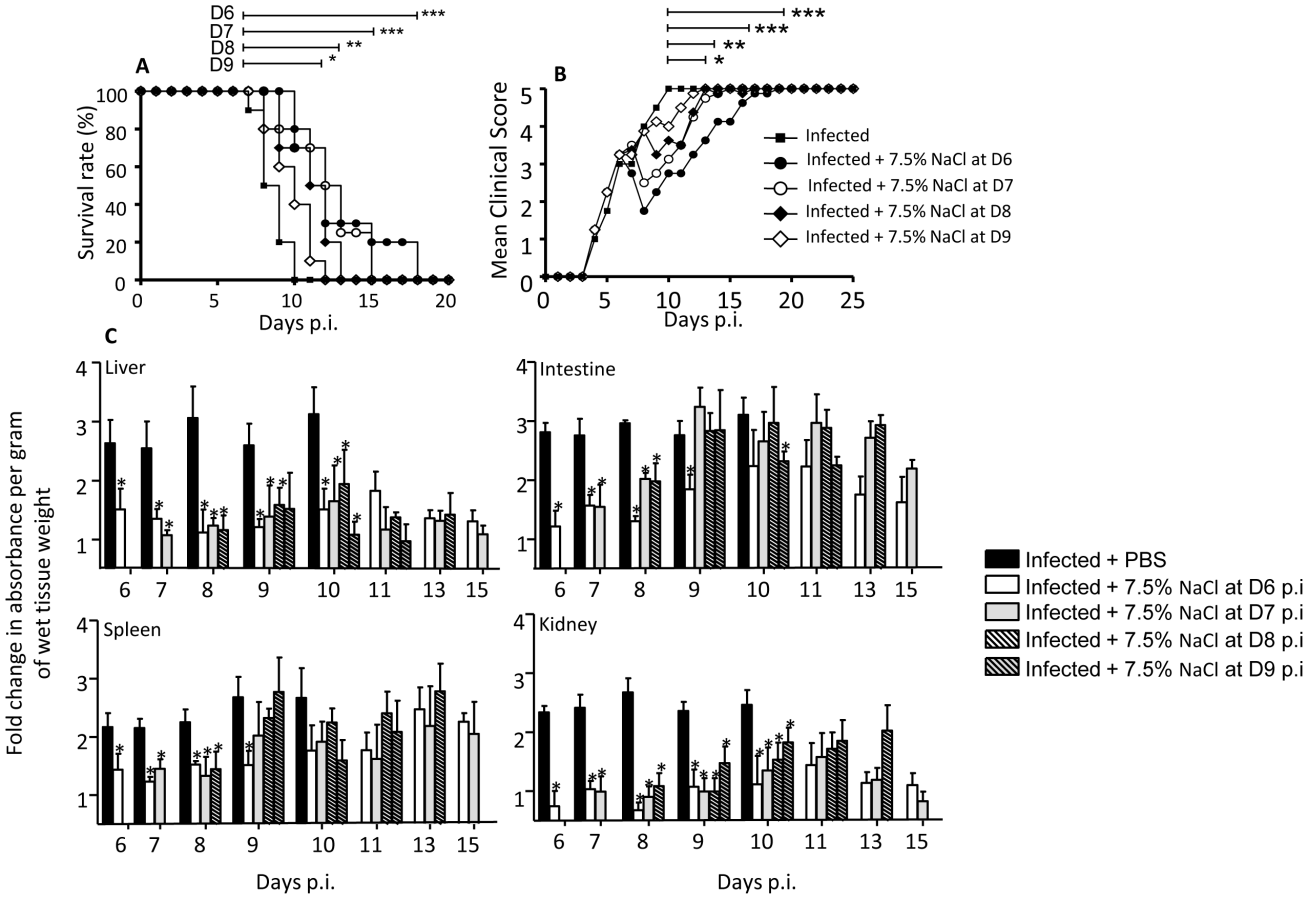
Survival rate, clinical score and vascular leakage in DENV-infected mice treated once with 7.5% NaCl at day 6, 7, 8 or 9 post-infection. Groups of 8–9 week-old AG129 mice were sc. infected with D2Y98P-PP1 strain. At day 6, 7, 8 or 9 p.i., they were iv. administered with a single bolus (4 ml/kg body weight) of sterile 7.5% NaCl (HTS). (A) The survival rate (n = 10). (B) Clinical scores (n = 10); see legend of Fig. 1. (C) Vascular leakage. At the indicated time points post-HTS treatment, mice (n = 5) were euthanized and Evan’s blue extravasation assay was performed. Results are expressed as fold change compared to uninfected untreated control mice. *p<0.05.

Day 6 p.i. corresponds to the onset of detectable vascular leakage in the DENV-infected mice [20]. To test the efficacy of a single bolus of 7.5% NaCl administered at a later stage when vascular leakage is more pronounced, DENV-infected mice were treated at day 7, 8 or 9 p.i. The data clearly showed that the effect of HTS on the survival and clinical manifestations was less pronounced as the time of administration is delayed (Fig. 3A&B). The extent of vascular leakage reduction in the liver and kidneys was comparable for all the treated groups regardless of the time of administration (Fig. 3C). In contrast, reduction of vascular leakage in the intestine and spleen was more effective with HTS treatment administered at day 6 p.i. whereby reduction was observed over a longer period of time post-treatment (Fig. 3C).

### Effect of HTS on the Virus Titers, Organ Damage and Hematological Parameters

**Figure 4:**
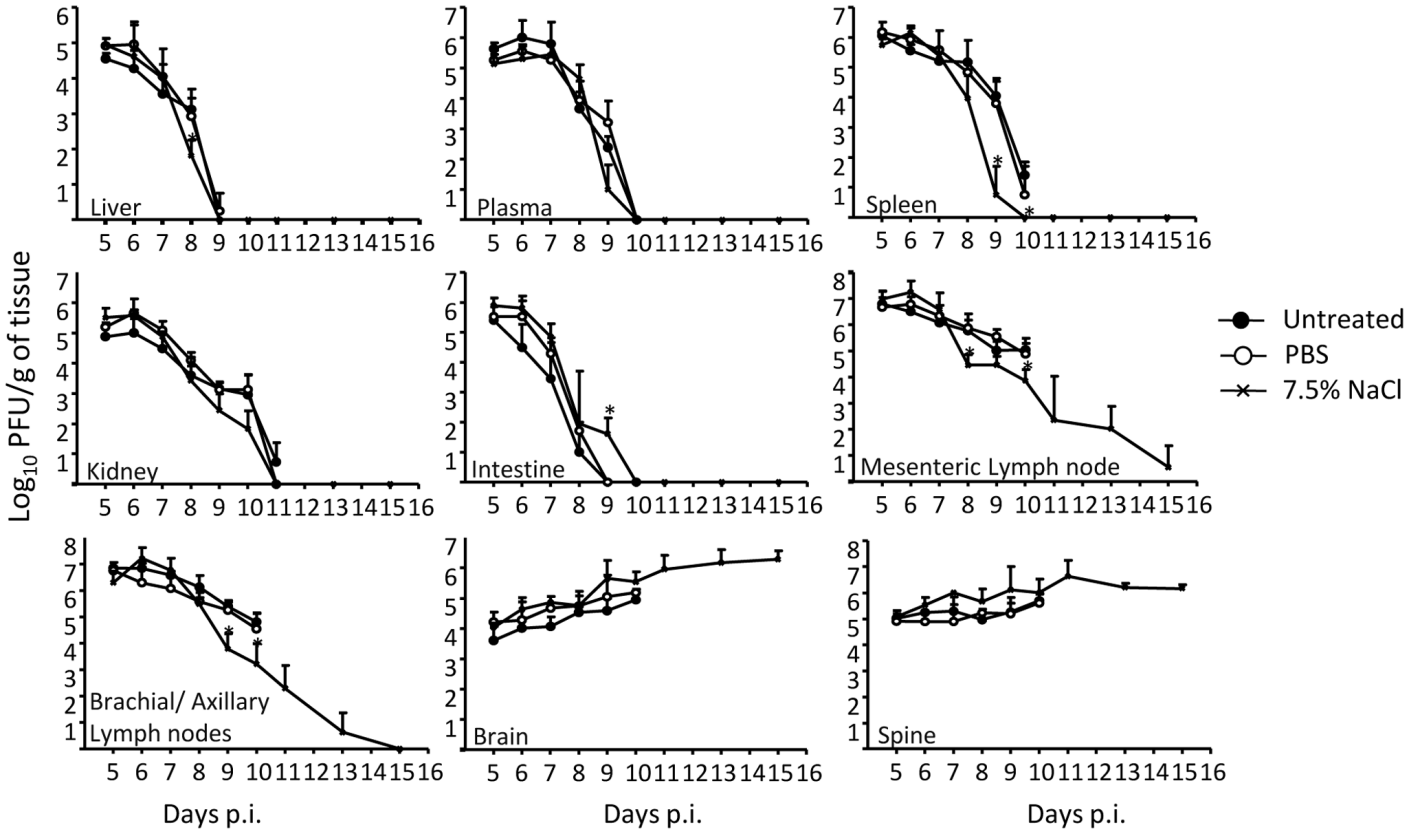
Virus titers in DENV-infected mice treated once with 7.5% NaCl at day 6 post-infection. Groups of 8–9 week-old AG129 mice were sc infected with D2Y98P-PP1 virus and at day 6 pi., they were left untreated (black circle) or they were treated iv. with a single bolus (4 mL/kg body weight) of 7.5% NaCl (black cross) or PBS (open circle). At the indicated time points post-infection, five animals per group were bled and euthanized. After perfusion with PBS, various organs were harvested as indicated. Virus quantification in the serum and organs was determined by plaque assay. Results are expressed for each organ/serum sample as the mean ± SD derived from five individual mice per time point.

To further characterize the effects of a single bolus of HTS given at day 6 p.i., the viral titers were determined in HTS-treated mice and compared to PBS-treated or untreated mice. The data indicated that HTS treatment generally did not affect the kinetic of the virus dissemination and replication (Fig. 4). Noticeably, probably as a result of prolonged survival time of the HTS-treated animals, the virus particles were totally cleared from the mesenteric and brachial/axillary lymph nodes. In contrast, sustained and high virus titers were found in the brain and spinal cord of the HTS-treated animals (Fig. 4).

**Figure 5:**
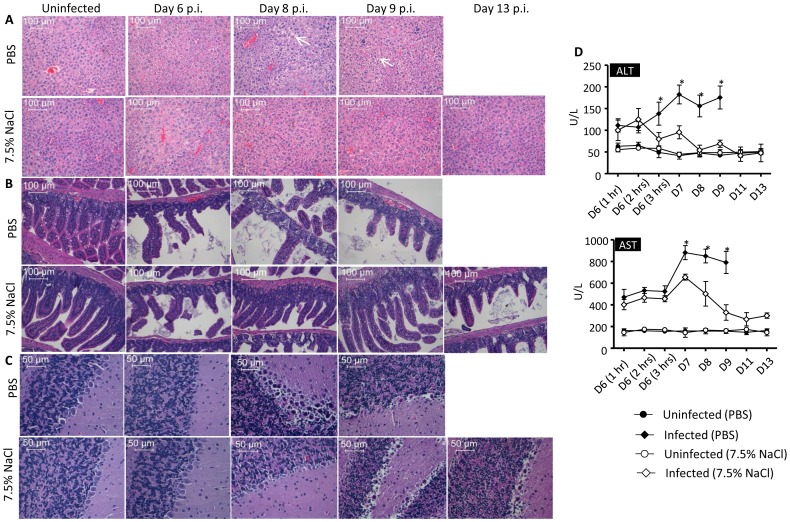
Histology examination and liver enzymes from DENV-infected mice treated once with 7.5% NaCl at day 6 post-infection. Groups of 8–9 week-old AG129 mice were left uninfected or were sc infected with D2Y98P-PP1 virus and at day 6 pi., they were treated with a single bolus (iv; 4 mL/kg body weight) of 7.5% NaCl (HTS) or PBS as indicated. At the indicated time points post-infection, the animals were euthanized and their liver (A), intestines (B) and brain (C) were harvested, fixed and processed for H&E staining. Observations were made at x200 (A, B) and x400 (C) magnification. Representative sections from 3 individual mice are shown. (D) Levels of serum alanine (ALT) and aspartate (AST) transaminases were monitored over time post-infection upon treatment with HTS or PBS as indicated. Results are expressed as the mean ± SD of five individual mice per group per time point.

Histology analysis of key organs revealed that hepatic necrosis characterized by pyknotic nuclei and cytoplasmic vacuolation of hepatocytes was much less pronounced in the HTS-treated animals compared to PBS-treated mice (Fig. 5A). Likewise, the serum levels of alanine (ALT) and aspartate (AST) transaminases were significantly lower in the HTS-treated mice compared to the PBS-treated group and eventually reached baseline level by day 8 and 11 p.i. respectively indicating reduced liver inflammation (Fig. 5D). The intestines from the HTS-treated mice also displayed less damage than the PBS-treated group, although villi detachment and disintegration were not totally prevented (Fig. 5B). Finally, vacuolation of the Purkinje cell layer was clearly observed in the cerebellum from both infected animal groups, indicative of brain tissue damage (Fig. 5C).

**Figure 6:**
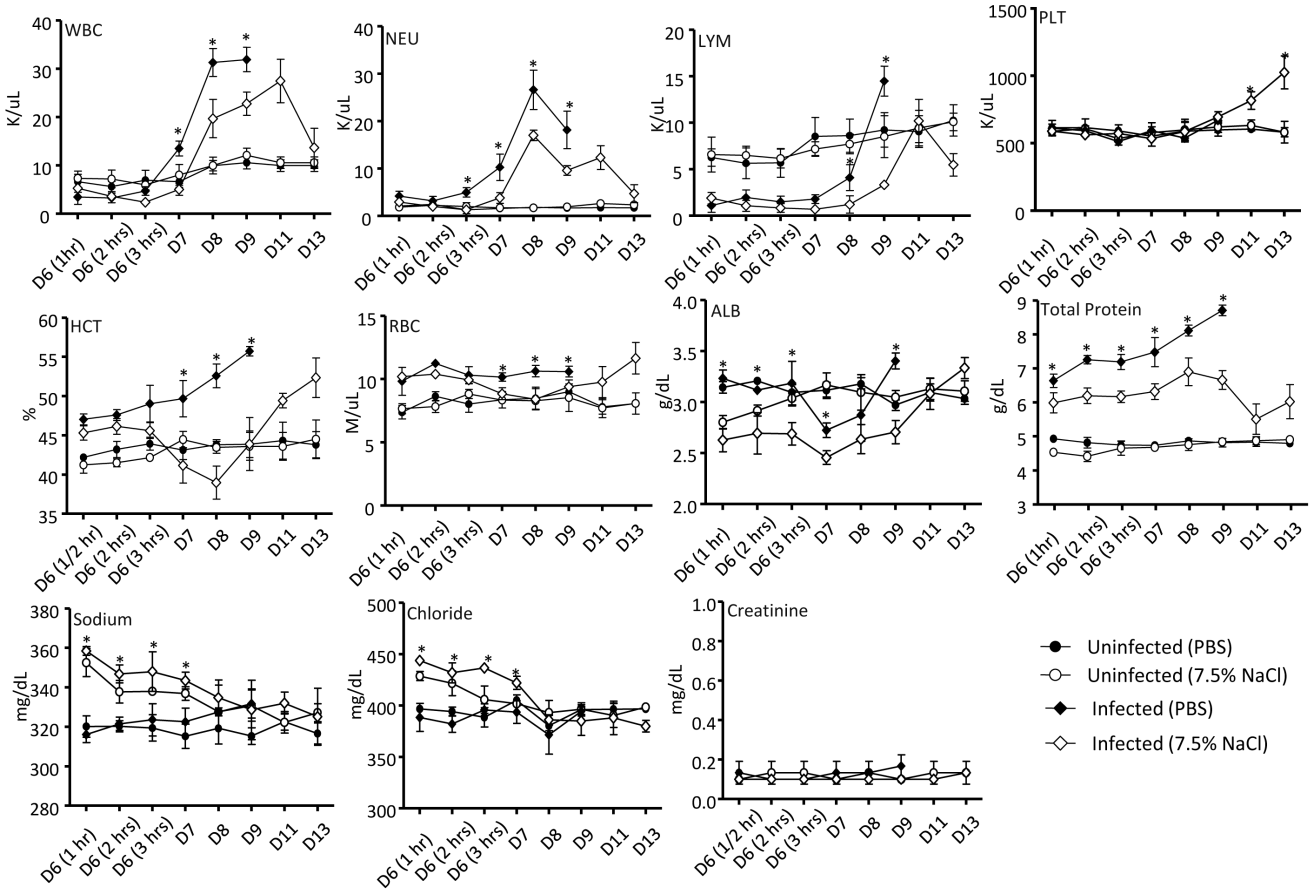
Hematology in DENV-infected mice treated once with 7.5% NaCl at day 6 post-infection. Groups of 8–9 week-old AG129 mice were left uninfected or were sc infected with D2Y98P-PP1 virus and at day 6 pi., they were treated with a single bolus (iv; 4 mL/kg body weight) of 7.5% NaCl (HTS) or PBS as indicated. At the indicated time points post-infection, 5 mice per group were euthanized and bled for hematology analysis. Results are expressed as the mean ± SD of the 5 individual measurements. Legend: WBC, white blood cells; NEU, neutrophils; LYM, lymphocytes; PLT, platelets; RBC, red blood cells; HCT, hematocrit; ALB, albumin.

Analysis of the blood parameters revealed that the white blood cells (WBC), neutrophils (NEU) and lymphocytes (LYM) counts were generally lower in the HTS-treated infected mice than those measured in the PBS-treated infected animals, although the counts remained higher than those measured in the uninfected controls (Fig. 6). Platelets (PLT) counts were comparable in all the groups, except at day 11 and 13 p.i. where higher PLT counts were measured in the HTS-treated infected animals (Fig. 6). Hematocrit (HCT) and red blood cell (RBC) counts were significantly lower in the HTS-treated infected mice compared to the PBS-treated infected group. Albumin and total protein concentrations were significantly lower in the HTS-treated infected animals than in the PBS-treated infected animals. As for the electrolytes, the level of sodium and chloride ions were transiently increased in the HTS-treated groups (infected and uninfected) compared to the levels measured in the PBS-treated mice but were back to baseline levels within 24 to 48 hrs post-treatment (Fig. 6). Finally, the levels of creatinine were comparable in all the animal groups suggestive of normal kidney function.

### Production of Inflammatory and Non-inflammatory Mediators in HTS-treated Mice

**Figure 7:**
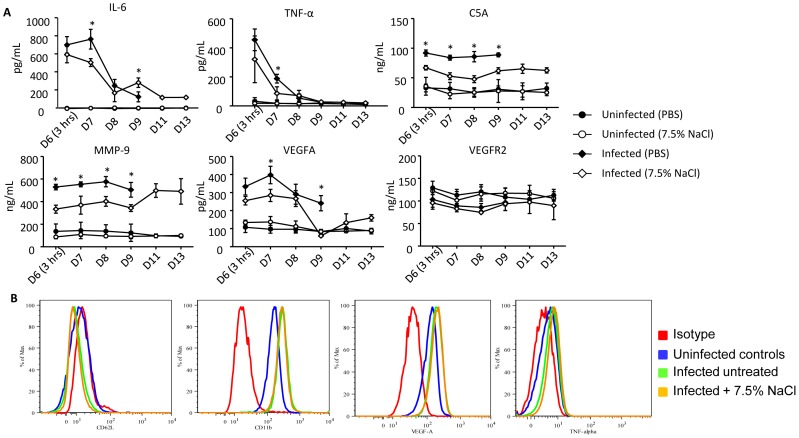
Serum levels of soluble mediators and neutrophil activation in DENV-infected mice treated once with 7.5% NaCl at day 6 post-infection. Groups of 8–9 week-old AG129 mice were left uninfected or were sc infected with D2Y98P-PP1 virus and at day 6 pi., they were treated with a single bolus (iv; 4 mL/kg body weight) of 7.5% NaCl (HTS) or PBS as indicated. (A) At the indicated time points post-infection, 5 mice per group were euthanized and bled for measurement of IL-6, TNF-α, complement protein C5a, MMP9, VEGFA and its receptor VEGFR2. Results are expressed as the mean ± SD of five individual samples per time point. (B) At 24 hrs post-HTS or PBS treatment, 5 mice per group were bled and FACS analysis was performed to detect surface expression of CD62L and CD11b, and intracellular production of TNF-α and VEGFA.

The serum levels of IL-6, TNF-α, metalloprotease 9 (MMP9), C5a complement protein, vascular endothelial growth factor A (VEGFA) and its receptor VEGFR2 were measured in the PBS- or HTS-treated DENV infected mice. The results showed that the systemic concentration of IL-6 and TNF-α was significantly lower in the HTS-treated group at day 7 p.i., i.e one day post-treatment compared to those that received PBS (Fig. 7A). Serum levels of C5a, MMP9, and VEGFA were also significantly lower in the HTS-treated infected animals compared to the PBS-treated infected group and these lower levels were sustained for at least 3 days post-treatment (Fig. 7A). In contrast, there was no significant difference in the concentration of VEGFR2 in all the animal groups (Fig. 7A). Together, these results indicated that the systemic levels of key inflammatory and non-inflammatory mediators proposed to play a role in DEN-associated vascular permeability, are significantly decreased upon HTS treatment in DENV-infected animals.

### HTS does not Alter the Neutrophil Activation Status

Previous literature reported that HTS reduces the expression of neutrophil adhesion molecules CD11b and CD62L, thereby impairing their activation status which may reduce inflammation and organ damage [9], [10], [13]. However, FACS analysis revealed that the activation status of the circulating neutrophils in the HTS-treated DENV infected mice was similar to that observed in the non-treated infected controls as evidenced by comparable levels of expression of CD62L and CD11b (Fig. 7B). In addition, intracellular staining of neutrophils indicated that their production level of VEGF-A and TNF-α is not affected by HTS treatment (Fig. 7B).

**Figure 8:**
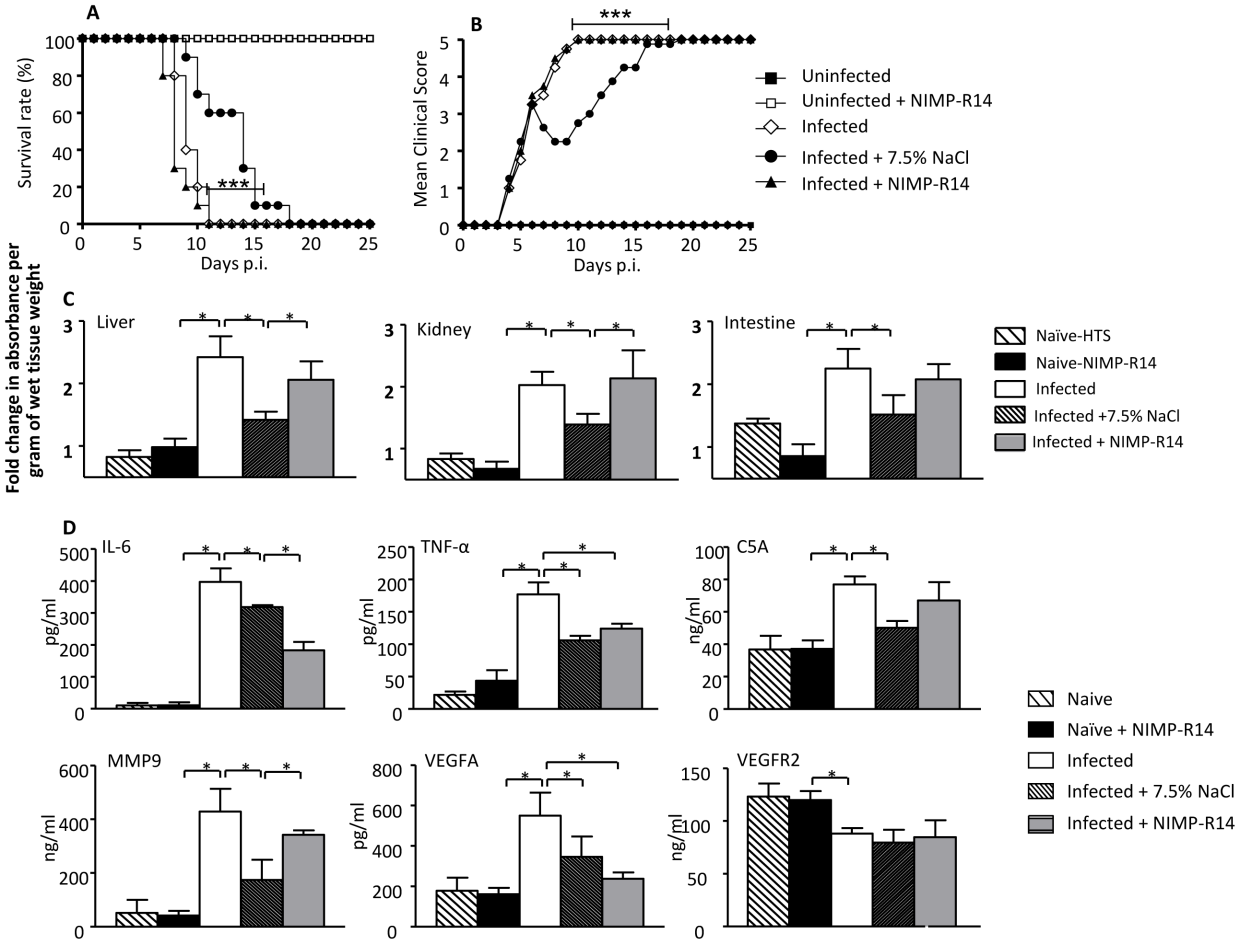
Effect of neutrophil depletion in DENV-infected mice. Groups of 8–9 week-old AG129 mice were left uninfected or were sc infected with D2Y98P-PP1 virus. At day 5 and 6 p.i., groups were ip. injected with NIMP-R14 antibodies. A control group was iv. injected with a bolus of 7.5% NaCl (4 ml/kg) at day 6 p.i. (A) Survival rate (n = 10). (B) Clinical score (n = 10) See legend of Fig. 1. (C) Vascular leakage. At day 7 p.i., mice (n = 5) were euthanized and Evan’s blue extravasation assay was performed. Results are expressed as fold change compared to uninfected untreated control mice. *, p<0.05. (D) Serum levels of soluble factors. At day 7 p.i., mice (n = 5) were euthanized and the serum levels of IL-6, TNF-α, C5a, MMP9, VEGFA and VEGFR2 were measured. The results are expressed as the average of 5 individual samples ± SD. *p<0.05.

To further investigate the role of neutrophils during DENV infection, DENV-infected animals were treated with NIMP-R14 antibody that selectively depletes the neutrophil population [21]. FACS analysis of the circulating immune cell populations confirmed that NIMP-R14 treatment significantly reduced the neutrophil concentration to a level comparable to that obtained upon HTS treatment (data not shown). However, NIMP-R14 treatment did not affect the survival rate and clinical score of the infected animals which were similar to that observed with the non-treated group (Fig. 8A&B). Neither did the antibody treatment result in any significant reduction of vascular leakage in the infected animals (Fig. 8C). The systemic levels of inflammatory and non-inflammatory mediators were measured 24 hrs post-depletion and showed decreased concentrations in IL-6, TNF-α and VEGFA, whereas the levels of MMP9 and C5a were not significantly different from the non-treated infected group (Fig. 8D).

Together, these observations thus indicate that neutrophils contribute to the serum levels of IL-6, TNF-α and VEGFA but are not directly responsible for DEN-associated vascular leakage. The data also suggest that the lower neutrophil counts measured in HTS-treated animals do not account for the reduction of vascular leakage.

## Discussion

A number of hypertonic resuscitation studies have been reported and together with animal studies have demonstrated that HTS infusion in small volumes (3 to 6 mL/kg) rapidly improves cardiovascular and metabolic function through a combination of plasma volume expansion, systemic vasodilation, and improved myocardial performance [7], [22]. However, recent clinical trials have failed to demonstrate the superior efficacy of HTS over other fluid resuscitation strategies [23]–[25]. The heterogeneity in the trial designs, treatment regimen, read-outs, and the low number of patients enrolled for each study have precluded a definitive consensus and the debate remains largely open. As such HTS treatment has never been considered as a conventional intervention procedure and remains a therapeutic alternative that is carried out at the clinicians’ discretion and when the standard resuscitation approach, i.e administration of large volume of isotonic fluids fails or is contra-indicated.

Some safety concerns have also been raised over electrolyte imbalance upon HTS administration. Hypernatremia, hyperosmolality, hypokalemia, and hyperchloremia may indeed result in disorientation, confusion and seizure, and can increase the risk of renal failure, pulmonary edema and congestive heart failure [26], [27]. With these restrictions in mind, we showed in a mouse model of DSS that a single bolus of 7.5% NaCl at 4 mL/kg administered at the onset of detectable vascular leakage (i.e 6 days p.i.) led to a significant reduction of vascular leakage which was sustained for at least 3 days post-treatment. HTS concurrently stopped the diarrhea symptoms and delayed the animals’ death. This was accompanied by reduced tissue (intestine/liver) damage and restoration of the hepatic functions. Transiently elevated levels of sodium and chloride ions were expectedly measured in the HTS-treated animals and were back to basal levels within 24 to 48 hours post-treatment. Importantly, the levels of creatinine were found comparable in all the animal groups, an indicator of the absence of kidney damage and failure upon HTS treatment.

A single bolus of 7.5% NaCl administered at day 6 post-infection however reduced vascular leakage only transiently (up to 3 days post-administration). In an attempt to sustain the vascular leak reduction over a longer period of time, 3 consecutive daily administrations were given (day 6–8). This treatment was found more effective than a single bolus at reducing vascular leakage and at delaying the animals’ death. However, signs of kidney impairment indicated that such regimen is not advisable. We have also explored the injection of a second bolus of HTS 6 days after the first bolus (day 12 pi.) when vascular leakage is back to levels comparable to the untreated infected animals. This second bolus allowed reducing vascular leakage and delayed further the animals’ death compared to the animal group treated only once at day 6 pi.(data not shown). However, the safety aspect of this 6-days apart double dose regimen remains to be investigated.

Fluid management of dengue patients consists of the intravenous administration of isotonic fluids (RL and others) at various rates and over a several hours period. Such intervention procedure is not possible to perform in a mouse model. We have thus not been able to compare in our experimental set up HTS treatment with the standard care approach. In our study, the PBS control allows demonstrate that the effects observed are due to the hypertonic nature of the HTS suspension rather than the volume of liquid injected. In addition to a rapid correction of fluid loss, the advantage of HTS versus isotonic fluid is the administration of a much smaller volume which avoids the risk of excessive fluid accumulation in tissues. Therefore, although transient in our mouse model, HTS treatment may represent a faster therapeutic intervention compared to isotonic fluid for a rapid correction of fluid loss, in particular in severe shock cases. Combining a single bolus (small volume) of HTS for a rapid correction of fluid loss followed by isotonic fluid administration for maintenance may be worth considering further.

The actual cause of death in the DENV-infected AG129 mice (HTS treated or untreated) has remained unclear. Our study showed that a single bolus of HTS delayed the animals’ death but led to increased viral titers in the CNS. It may thus be possible that mice die as a result of CNS damage. However, as mentioned above, we observed that a second bolus of HTS given at day 12 post-infection reduced vascular leakage and further delayed the animals’ death with a median of 6 days difference between mice treated once (day 6 pi) and mice treated twice (day 6 and 12 pi.) (data not shown). Furthermore, in this two doses-treated group, we found a clear correlation between moribund state and increased vascular leakage whereby moribund animals presented higher vascular leak compared to non-moribund animals. These observations thus support that instead of CNS involvement due to increased viral loads, vascular leakage is the primary cause of death in the infected animals. Meningitis and CNS involvement have rarely been reported in DEN patients who are mainly immunocompetent, and this feature is one of the weaknesses of the AG129 mouse model. A recent study has indeed shown that absence of the type I interferon pathway in these mice is responsible for the viral spread to the CNS [28].

The current paradigm of the mechanisms responsible for DEN associated vascular leakage involves a variety of soluble mediators known to affect vascular permeability, including pro-inflammatory cytokines and chemokines as well as non-inflammatory mediators [29], [30]. Here, lower levels of IL-6, TNF-α, MMP9, C5a, and VEGFA were measured in the HTS-treated mice, all of these factors have been found elevated in DEN patients and correlated with disease severity [31]. In addition to its role as a plasma volume expander through osmotic effect, increasing experimental evidence have been accumulating that demonstrate the immunomodulatory properties of HTS. Hypertonicity was indeed reported to reduce neutrophil activation and expansion of pro-inflammatory monocytes which correlated with lower levels of pro-inflammatory cytokines [10]. Consistently, we measured lower WBC and NEU counts, and lower systemic levels of IL-6, TNF-α and VEGFA in the HTS-treated infected mice. However, the activation status of circulating neutrophils and their production levels of TNF-α and VEGFA were not affected by HTS treatment. Thus, together these observations suggested that in DENV-infected mice, HTS reduced the counts of circulating immune cells which correlated with lower systemic levels of key inflammatory and non-inflammatory mediators, thereby possibly translating into reduced vascular permeability. Whether these lower levels only result from the mobilization of fluid into the intravascular space as a dilution effect and/or are accounted for by the immunomodulatory properties of HTS remains to be elucidated. Furthermore, neutrophil depletion revealed that neutrophils do not play a critical role in DEN-associated vascular leakage, and that the ability of HTS to reduce vascular leakage is unlikely to be mediated by its effect on the neutrophil counts. Further work is needed to identify whether the effect of HTS on other immune cells plays a role in its protective efficacy against DEN associated vascular leakage.

In conclusion, our study demonstrates that administration of a hypertonic solution effectively but transiently reduces vascular leakage in a mouse model of severe dengue infection. Our findings are consistent with the conclusions of a recent clinical trial conducted in Indonesia in DSS children [16]. The study indicated significantly lower plasma levels of sVCAM-1 as a marker of endothelial leakage, and faster capillary refill time (CRT) recovery as a marker of microcirculatory perfusion, in the DSS children who received Hypertonic Sodium Lactate (HSL) compared to DSS children treated with Ringer Lactate. The study concluded that HSL administration was suited for early fluid resuscitation in DSS.
